# Characterization of Engineered Actin Binding Proteins That Control Filament Assembly and Structure

**DOI:** 10.1371/journal.pone.0013960

**Published:** 2010-11-12

**Authors:** Crista M. Brawley, Serdar Uysal, Anthony A. Kossiakoff, Ronald S. Rock

**Affiliations:** Department of Biochemistry and Molecular Biology, The University of Chicago, Chicago, Illinois, United States of America; University of Birmingham, United Kingdom

## Abstract

**Background:**

Eukaryotic cells strictly regulate the structure and assembly of their actin filament networks in response to various stimuli. The actin binding proteins that control filament assembly are therefore attractive targets for those who wish to reorganize actin filaments and reengineer the cytoskeleton. Unfortunately, the naturally occurring actin binding proteins include only a limited set of pointed-end cappers, or proteins that will block polymerization from the slow-growing end of actin filaments. Of the few that are known, most are part of large multimeric complexes that are challenging to manipulate.

**Methodology/Principal Findings:**

We describe here the use of phage display mutagenesis to generate of a new class of binding protein that can be targeted to the pointed-end of actin. These proteins, called synthetic antigen binders (sABs), are based on an antibody-like scaffold where sequence diversity is introduced into the binding loops using a novel “reduced genetic code” phage display library. We describe effective strategies to select and screen for sABs that ensure the generated sABs bind to the pointed-end surface of actin exclusively.

**Conclusions/Significance:**

From our set of pointed-end binders, we identify three sABs with particularly useful properties to systematically probe actin dynamics: one protein that caps the pointed end, a second that crosslinks actin filaments, and a third that severs actin filaments and promotes disassembly.

## Introduction

The actin cytoskeleton found in all eukaryotes defines many of the essential mechanical properties of the cell. The balance of forces on actin filaments controls the overall shape of the cell and its ability to adhere to substrates and neighboring cells. Moreover, actin filaments are dramatically remodeled in protrusive areas at the leading edges of migrating cells, and at the cleavage furrow during cytokinesis [1]. Actin filaments (F-actin) are constructed from the polymerization of individual 43 kDa globular monomers (G-actin) into a two-start helix with both lateral and longitudinal interactions between monomers [2]. The F-actin filament is polar, with distinct ends known as the barbed and pointed ends. These two ends maintain distinct polymerization and depolymerization rates, a property that requires the hydrolysis of bound ATP after polymerization [3]. Polymerization occurs rapidly after a nucleus of ∼3 actin monomers is formed [2]. Since actin is an abundant cellular protein, its ability to form filaments is under tight cellular control. Indeed, over a hundred distinct actin binding proteins (ABPs) modulate the properties of actin to establish filaments at precise locations, while preventing spontaneous assembly throughout the cell [4]. Examples of ABP function include the nucleation of filament formation in response to upstream signals, capping filaments to prevent elongation from the barbed end, depolymerization or severing of filaments, modulation of filament stiffness, bundling or crosslinking filaments into higher order assemblies, and sequestering actin monomers to block spontaneous nucleation.

Given the rich and complex behavior of actin and ABP systems, we set out to determine the feasibility of generating novel classes of artificial ABPs that could mimic the functions of some of the natural ABPs through a defined mode of action. We reasoned that many ABPs work through effects produced on binding at either the barbed or pointed end of actin filaments. It appears that a vast majority of structurally characterized ABPs bind to the barbed end, while relatively few are known to target the pointed end [5], [6]. Known pointed end binders include DNase I [7], tromodulin [8], [9], Arp2/3 [10], and emerin [11]. In addition, certain WH2 domain proteins make extensive contacts with actin that reach the pointed end [12]. Two of these pointed-end binding proteins work as part of a larger complex, namely tropomyosin/tropomodulin, and Arp2/3. Our challenge was to produce new artificial capping proteins that would bind to the pointed end of actin filaments and block polymerization.

To this end, we generated a set of “synthetic Antigen Binders” (sABs) using a phage-display combinatorial library that acted like novel monomeric pointed-end ABPs. This phage display library uses a reduced genetic code to introduce sequence and conformational diversity into the antigen recognition loops of an antibody-like (Fab) scaffold [13], [14]. Since the sABs are selected under exquisitely controlled *in vitro* conditions, the whole process overcomes many of the challenges associated with conventional antibody production in laboratory animals. A key advantage is that the sABs are guaranteed to recognize native, folded targets. With carefully designed selection strategies, sABs are generated that bind to a specific epitope on the target, stabilize specific target conformations, and identify intact complexes instead of the individual components. All of these design features may be used to target specific regions of actin and to mimic features of natural ABPs, in this case specific pointed end binders.

Here we describe the generation and characterization of a series of sABs specifically designed to bind in a regio-selective manner at the pointed end of actin. The work here expands on our earlier preliminary report of these sABs as part of a project to develop a live-cell receptor-mediated delivery system [15]. Surprisingly, the sABs described here not only sequester G-actin and block polymerization from the pointed end, but they also bind to F-actin filaments and alter filament behavior. We demonstrate examples of sABs that cap the pointed end of filaments, sABs that crosslink filaments into bundles, and sABs that sever actin filaments. The ability to generate highly specific and functional artificial ABP mimics opens up the possibility to manipulate features of the actin cytoskeleton in much more controlled ways than is now possible with natural ABPs. Consequently, we imagine that these sABs will be particularly useful reagents for the cytoskeletal community. These sABs may be combined with barbed-end capping proteins to generate actin filaments of a defined length, or to arrange filaments in defined patterns and orientations on substrates. These organized actin structures would be particularly useful for *in vitro* mechanical and transport studies.

## Results

### Library sorting for the pointed end of actin

**Figure 1:**
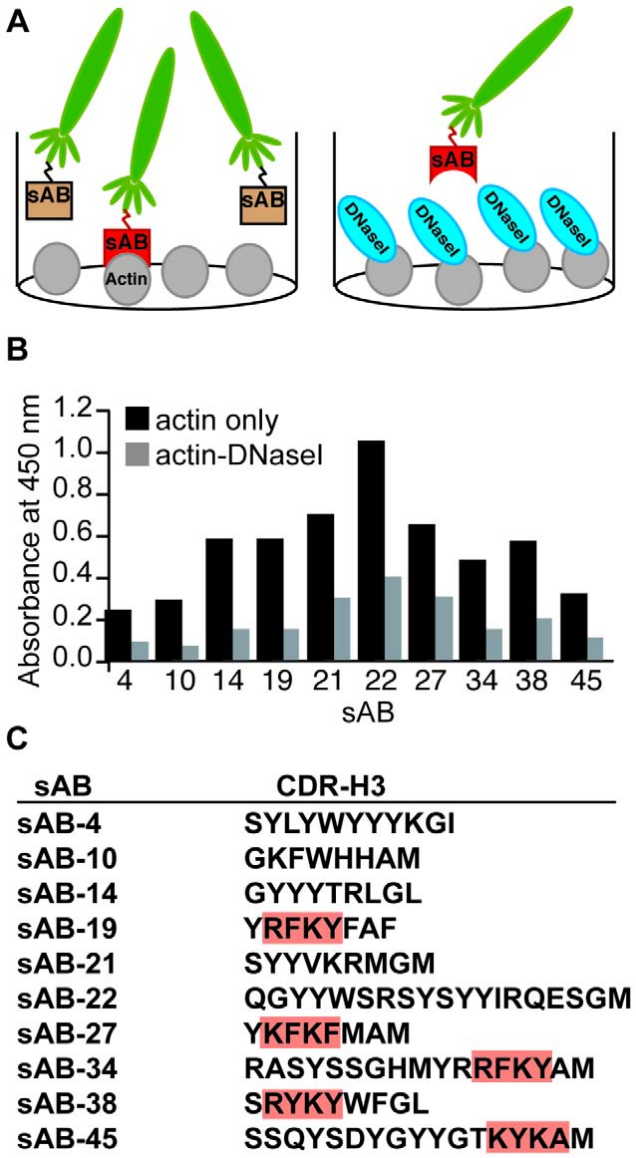
Scheme for sAB creation. (A) A cartoon of the phage ELISA experimental scheme. Phages displaying single antibody fragment are shown in green. Actin and DNase I are labeled. Actin is immobilized in the well, treated with ± DNase I, and exposed to phages. Total bound phage quantity is recorded in the ELISA signal. (B) The ELISA signals for the ten unique sABs, ± DNase I. (C) CDR-H3 sequences of the ten unique actin binders.

We used a previously described restricted amino acid diversity library to generate antibodies to the pointed end of actin [13], [14]. The library sorting protocol is similar to the approach used to target membrane proteins [13], [14]. To target the pointed end of actin, we used the following two-part scheme. First, a reactive free cysteine residue (residue 374) at the barbed end of actin was biotinylated to allow actin to be immobilized on a bead surface during the phage display sorting. We reasoned that immobilization via the barbed end should bias selection towards the pointed end, since the pointed end would face away from the surface of the bead and be more accessible to the phage. Second, we screened the pool from the first stage using a competitive phage ELISA, to identify genuine pointed-end binders. DNase I is known to bind to the pointed end of G-actin monomers but not well to F-actin filaments. We used this feature of DNase I to identify phage binders that are captured by actin alone, but not the DNase I: actin complex (Fig. 1ab) [16]. These phage binders likely bind at the DNase I: actin interface.

Seventeen of the tested phage clones exhibited a decrease in ELISA signal of more than twofold upon DNase I competition. These were sequenced yielding ten unique sABs (Fig. 1c). Remarkably, four of these ten contained a regular four-residue sequence motif ([RK][YF][RK][YF]) in the complementarity determining region (CDR)-H3 loop. This sequence may represent a common actin-binding motif, as we estimate that the probability of this sequence motif appearing in four out of ten clones by chance alone is extremely remote (P∼10^−7^, see Supporting Information S1). Initial characterization of the binders by pull-down assays and gel filtration chromatography identified three of the binders, sAB-4, sAB-19 and sAB-27 as suitable candidates for further work (see the chromatogram in Fig. S1). Direct measurement of K_d_s for these sABs by surface plasmon resonance was problematic due to considerable noise that remained even when actin alone was examined. We suspect that this noise is due to the dynamic nature of the actin filaments. However, based on our experience of profiling over one hundred protein targets, we find an average K_d_ of approximately 10 nM when we produce sABs using 10 nM target in the final selection round [13], [14] (see Materials and Methods).

### Pointed end capping by sAB-19

**Figure 2:**
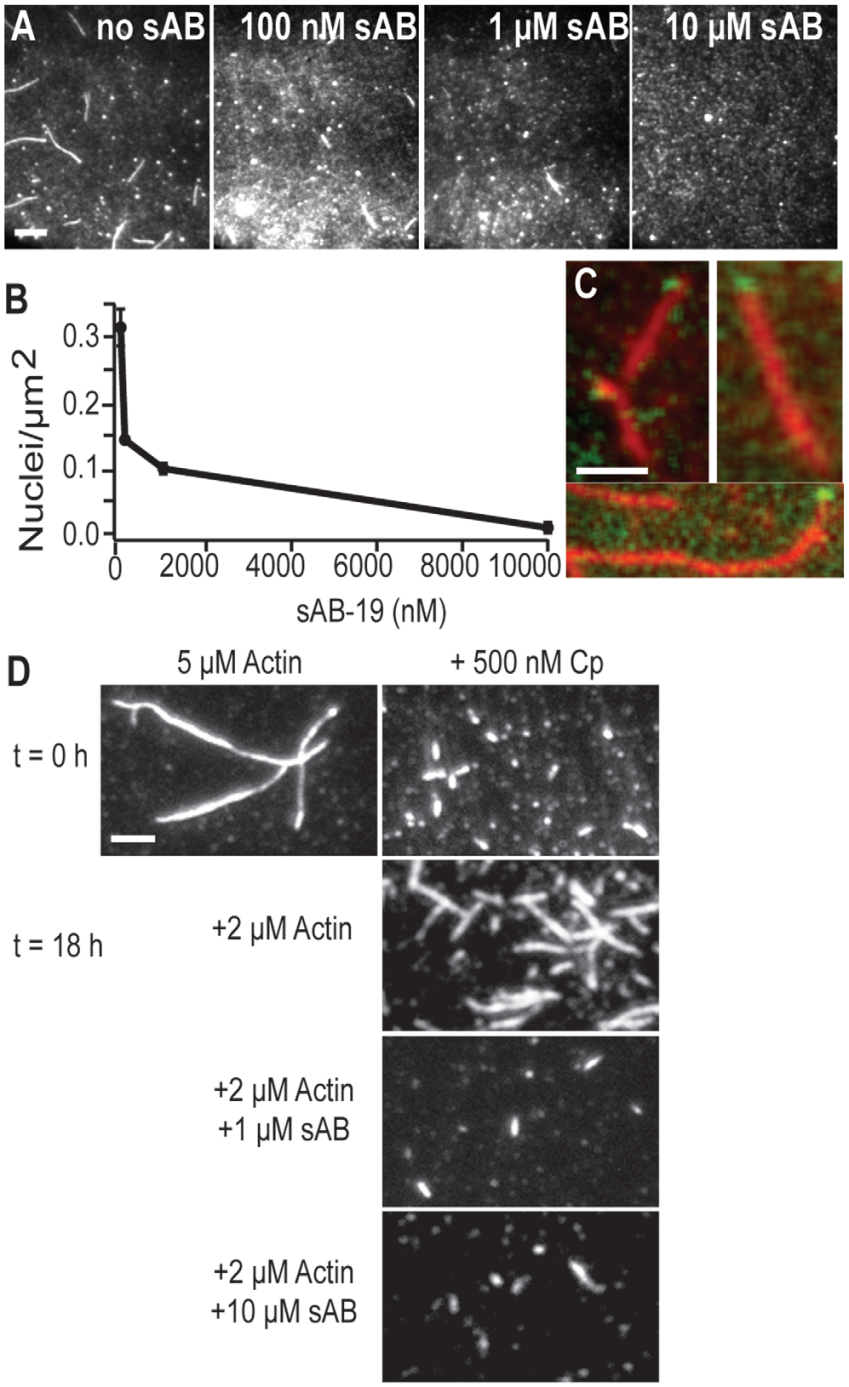
sAB-19 caps the pointed end of actin. (A) Visualization of actin nuclei present in TIRF microscopy after 5 minutes of polymerization. We counted only oblong objects as nuclei. In the absence of sAB-19, we observe more actin nuclei, and nuclei decrease as the sAB-19 concentration is increased. (B) Quantification of the number of actin nuclei found after 5 min as the sAB-19 concentration is increased. (C) TMR-labeled actin filaments (red) in the presence of Cy5-labeled sAB-19 (green). The sAB-19 binds to one end of the actin filaments. (D) Seeded polymerization assay in the presence of mouse capping protein (Cp). Actin polymerized in the absence of capping proteins produces long filaments (upper left). F-actin was polymerized with Cp overnight to generate very short actin seeds capped on the barbed end and exposed to polymerization on pointed end (upper right). After the seeds were generated, free G-actin (33% TMR) under polymerization conditions was added along with sAB-19 (0, 1, and 10 µM). Additional G-actin allows further polymerization off the pointed end of actin, generating longer Cp-seeded actin. However, the addition of sAB-19 caps this pointed end and blocks polymerization, leaving the short seeds and confirming that sAB-19 binds to the pointed end of actin.

To characterize the interaction of sAB-19 with actin, we performed a polymerization assay in the presence of sAB-19 and assessed the outcome using total internal reflection fluorescence (TIRF) microscopy (Fig. 2ab). In this assay, we allowed partially TMR-and-biotin-labeled G-actin to polymerize for 5 minutes before adding it to a flow cell for visualization. The number of actin seeds (distinct filaments) was counted for each experiment (Fig. 2b). As we increased the sAB-19 concentration from 0.1 to 10 µM, the number of seeds decreased accordingly, suggesting that sAB-19 inhibits actin nucleation in a concentration dependent manner. As a control, we examined the polymerization of actin in the presence of a random sAB, targeting maltose binding protein, and found that actin nucleation and actin polymerization was unaffected (Fig. S2). Thus, we conclude that the sAB scaffold itself does not contribute to the effects seen here.

To better visualize the sAB-19 and F-actin interaction, two-color TIRF was performed (Fig. 2c). TMR-F-actin was washed into a flow cell and allowed to adhere to the surface via neutravidin. Labeled sAB-19 (Cy5) was then washed into the same flow cell. Both actin and sAB-19 were imaged with TIRF microscopy (Fig. 2c), which showed that sAB-19 associates to one end of an actin filament. Quantization of this assay revealed 41 total sAB binding events on 209 actin filaments, with 18 filaments having multiple binding events at one end of the filament. No filaments yielded binding events at both ends, suggesting that sAB-19 prefers one end.

To verify that sAB-19 binding is directed specifically to the pointed end of the actin filament, we performed a seeded polymerization assay. Using TMR-labeled actin as above, we generated seeds that were blocked at the barbed end of actin using 500 nM capping protein. We adjusted the actin and capping protein concentrations to yield a uniform population of short seeds that were just visible in TIRF microscopy. After adding 2 µM additional actin to these seeds and allowing polymerization to occur for 18 h, we detected growth of actin filaments off of the pointed ends of the seeds (Fig. 2d). However, when we first added 1–10 µM sAB-19 to the capping-protein seeds, followed by the additional actin, we observed that the further polymerization of the seeds was entirely inhibited as would be expected if sAB-19 caps the pointed end.

### Additional modifications of filamentous actin by other artificial ABPs

#### a) Bundling of actin by sAB-27

**Figure 3:**
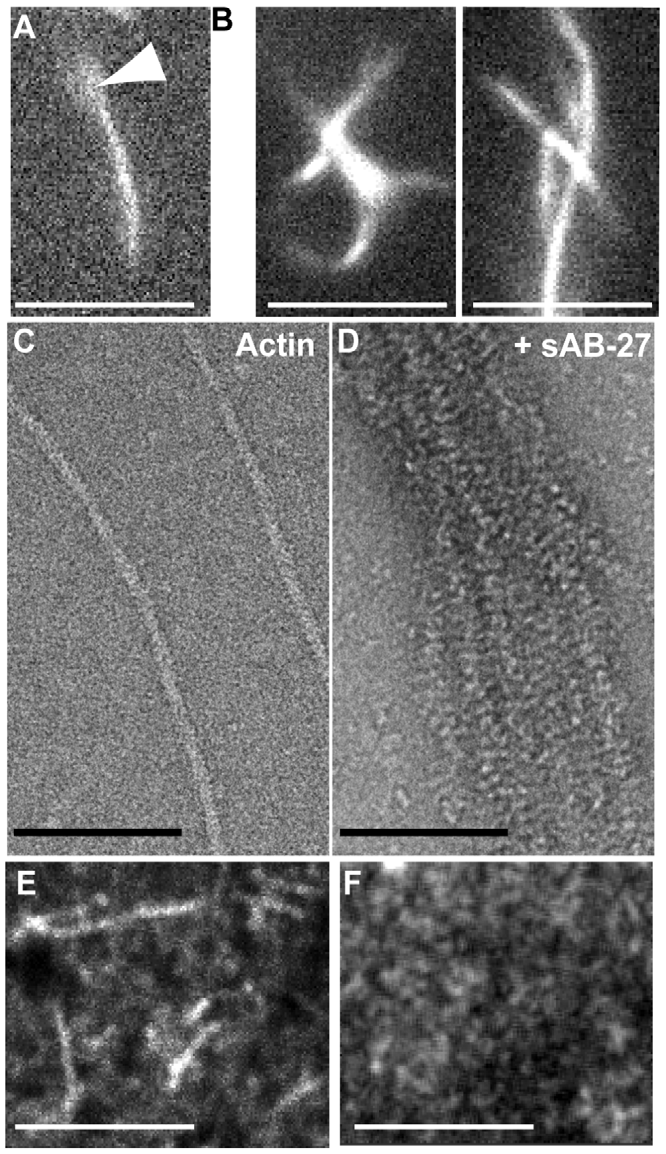
sAB-27 bundles actin filaments. (A) A single TMR-actin filament visualized in epifluorescence in solution. Arrowhead indicates an out of focus free end of F-actin. (B) TMR-actin filaments in solution with sAB-27, visualized in epifluorescence. Multiple filaments are crosslinked in the presence of sAB-27, and these crosslinked assemblies diffuse as a unit in solution. (C) Electron micrograph of single actin filaments. (D) Electron micrograph of actin filaments in the presence of sAB-27 (1 µM), revealing a similar crosslinking of actin filaments. (E) Nucleation assay of actin alone, after 5 min polymerization, visualized with TIRF microscopy. (F) Nucleation assay in the presence of 1 µM sAB-27 after 5 min. No polymerization is evident. Scale bar, 10 µm.

The other two sABs displayed behaviors that were remarkably distinct from sAB-19, even though they also targeted the pointed end of the actin filaments. Unlike sAB-4 and sAB-19, we found that sAB-27 displayed large fluctuations in pyrene fluorescence over time in the polymerization assays. These fluctuations could be due to heterogeneity in the bundled structures. We therefore rely on microscopy to analyze the direct effect of sAB-27 on actin. Adding sAB-27 to polymerized actin filaments induced extensive and apparently random crosslinking of the filaments into bundles. Using epifluorescence microscopy, we detected individual, freely moving filaments in solution in the absence of sAB-27 (Fig. 3a). However, upon addition of sAB-27, the single filaments disappeared and were replaced by “globular hairballs” of bundled filaments (Fig. 3b). Electron micrographs taken of F-actin and sAB-27 confirmed that the actin filaments were indeed bundled, revealing many parallel stretches of multiple overlapping filaments (Fig. 3cd). Once again, we can rule out contributions from the sAB antibody scaffold since a maltose binding protein sAB failed to crosslink actin filaments (Fig. S2). Interestingly, when actin polymerization was performed in the presence of sAB-27, no filaments were found in TIRF after 5 minutes (Fig. 3ef). This result suggests that sAB-27 may have an inhibitory effect on actin polymerization as well.

#### b) Severing of actin filaments by sAB-4

**Figure 4:**
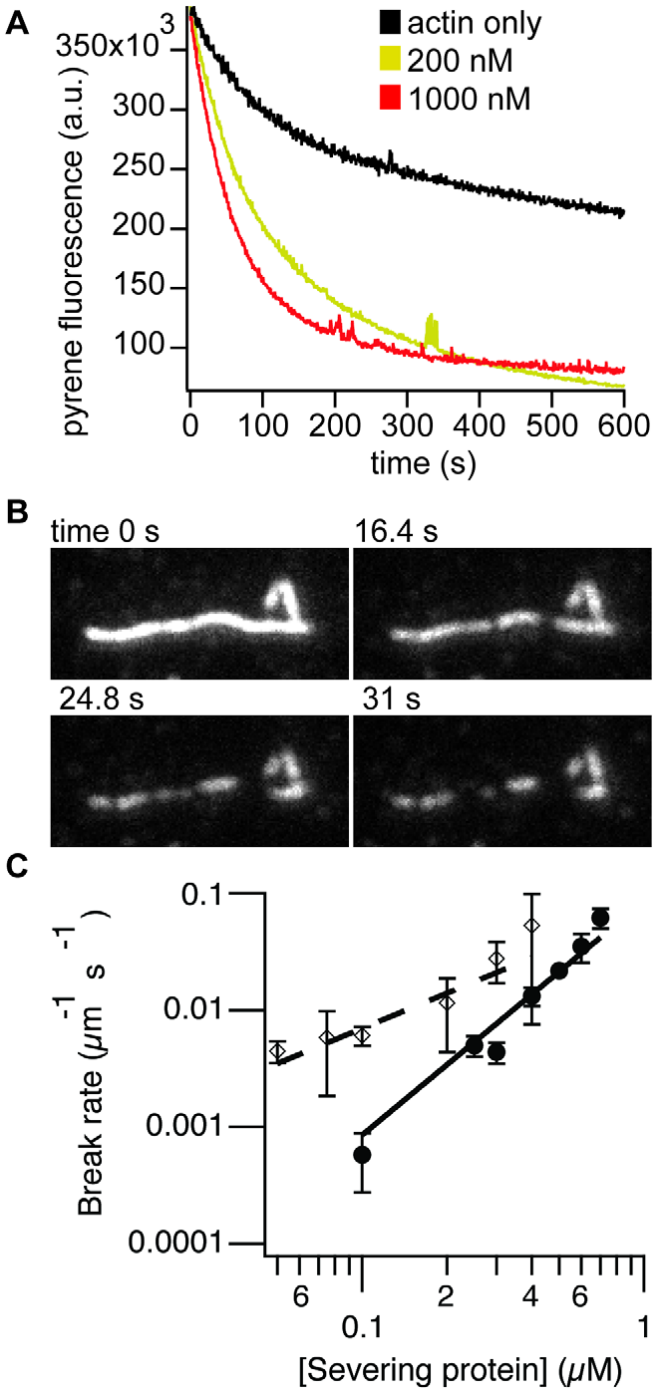
sAB-4 severs actin filaments. (A) Pyrene-actin depolymerization assay. Filaments are diluted to induce depolymerization. As the sAB-4 concentration increases, the pyrene fluorescence drastically decreases, indicating that sAB-4 enhances depolymerization. (B) TIRF images of an actin filament being severed over time by sAB-4 (0.4 µM). (C) Severing by sAB-4 is a second-order kinetic process. Break rate (µm actin^−1^ s^−1^) of actin filaments over increasing sAB-4 concentration (filled circles). The solid line is a second-power fit to the data (reduced χ^2^ = 3.6). For comparison, first-order severing by gelsolin (open diamonds) is also shown (reduced χ^2^ = 0.63).

Our final actin-binding protein sAB-4 acted as an actin-severing protein. Our initial evidence for this severing activity came from pyrene fluorescence measurements of the polymerized actin. A pyrene label on Cys374 served as a reporter of total filament quantity. When filaments were diluted, we observed gradual actin depolymerization. However, as we added increasing concentrations of sAB-4 and diluted the filaments, we observed more rapid depolymerization (Fig. 4a).

We further characterized sAB-4 using total internal reflection microscopy (TIRF) imaging, which allows us to observe the depolymerization of individual filaments in real time. In particular, we were interested in whether sAB-4 promotes depolymerization from the ends of the filaments, or if it severed filaments in the middle. We imaged long, directly-labeled tetramethylrhodamine (TMR)-actin filaments that were attached to the coverslip surface by widely-separated biotin-neutravidin links. We then added sAB-4 to the flow cell, which triggered the formation of multiple internal filament breaks (Fig. 4b), providing direct evidence that sAB severs the actin filaments. When we vary the sAB-4 concentration in these assays, we find that the break rate is apparently second-order in sAB-4 (Fig. 4c), suggesting that two sAB-4 binding events must occur for each filament break. The most likely explanation is that sAB-4 must bind to neighboring lateral or longitudinal sites on actin before severing occurs. However, one alternative interpretation of these data is that two severing events must occur for each apparent break, to allow the intervening segment of actin to diffuse away. To test this alternative, we used gelsolin in an identical filament-severing assay. Gelsolin severed filaments in an apparent first-order process (Fig. 4c), suggesting that we are able to detect individual severing events in this assay.

As with the other two sABs, when we polymerized actin in the presence of an equal quantity of sAB-4 no filaments were found in TIRF. Presumably, this result is due to the severing action of sAB-4 that would disassemble F-actin nuclei immediately upon polymerization, although sAB-4 may also sequester the G-actin monomers to block polymerization. We saw no evidence of severing with a sAB raised against maltose binding protein (Fig. S2).

## Discussion

### Generation of regio-selective sABs

Actin binding proteins (ABPs) are essential players in cytoskeleton dynamics by acting cooperatively to define the spatial separation and organization of filament branching, elongation and nucleation, as well as actin assembly and disassembly processes [4]. While the mechanisms through which individual ABPs function in context of their environments in the cytoskeletal system are a subject of intense investigation, many details remain obscure.

Given the numbers of ABPs, it seems surprising that such a large fraction of them appear to interact at a “hot spot” located at the barbed end. Some complexes of ABPs and actin have been crystallized, and the binding site for many of these ABPs appears to include a hydrophobic groove located between subunits 1 and 3 at the barbed end [5]. This groove is partially covered by the neighboring actin monomer, but still must be accessible in the actin filament. It is unclear why natural proteins seem to prefer this site over the rest of the exposed actin surface, other than the fact that this groove is an appropriate size and shape for a single hydrophobic α-helix. We note that had we used an unbiased selection for actin binders, many of our sABs may have instead bound to the barbed end like the characterized natural ABPs. However, our regio-selective phage display sorting schemes are quite effective at overcoming this apparent natural bias to bind the barbed end. Alternatively, the bias toward barbed-end binders may reflect the fact that polymerization occurs more rapidly at the barbed end [1], thus favoring the evolution of barbed-end binders to control filament growth at this end. Depolymerization, in contrast, is favored at free filament ends [17], so side binding and severing proteins are favored to generate additional free ends for depolymerization. Our *in vitro* selection processes are free of these constraints.

In general, the 10 sAB clones identified through this sorting process had high sequence and loop length diversity in CDR-H3. However, one striking feature was that in 4 of the 10 clones a common four-residue motif was observed: [R or K][Y or F][R or K][Y or F]. (A fifth clone, sAB-34, had a sequence KYKA). The probability of this pattern occurring by chance is highly remote, especially since R, K and F are populated in low abundance in the library. This suggests that this motif interacts with some specific pointed-end binding site in the actin molecule, although this needs to be confirmed by mutagenesis.

### Functions of artificial pointed-end ABPs

It was unknown whether the phage display generated sAB binders directed at the pointed end would all function predominantly as capping proteins or whether some would possess other properties. Based on the observations here, it is clear that this class of regio-specific sABs can manipulate actin dynamics in a variety of ways. Interestingly, if the sole objective was to identify sABs that cap the pointed end of actin and prevent polymerization, then only one of the ten sABs tested fits the criterion of an artificial capping ABP: sAB-19. However, an important by-product of the analysis was the finding that several of the other pointed-end binders produced distinct and extensive modulations of actin structure. For instance, sAB-4 effectively severs actin filaments much like cofilin and sAB-27 bundles actin filaments. Not discussed here are the properties of sAB-34, which depolymerizes actin filaments and sAB-10 that is a “side binder”, having no apparent effect on the filaments. As a side binder that induces no response on binding, sAB-10 is an effective imaging reagent for actin in live cell studies [15].

Taken together, these findings demonstrate that the pointed end of actin is very sensitive to subtle perturbations that can drive a highly diverse set of actin responses. It is unsurprising that actin dynamics requires a vast combination of ABPs to ensure tight functional regulation, especially during dramatic cytoskeletal remodeling events such as cytokinesis. Our results show that artificial ABPs having selectable characteristics can be generated using highly controlled phage display sorting strategies, opening up the possibility of manipulating actin dynamics in a rational fashion.

### Mode of action

Actin monomers are inherently dynamic and are believed to open or close their major cleft between subunits 2 and 4 in response to the bound nucleotide state [4]. The filaments undergo thermal breathing motions, with a variable rotation of ± 10° per actin monomer compared to the mean position [18]. This suggests the possibility that the binding of the sABs to the filaments may “lock-in” a set of different actin conformations at the pointed end, triggering associated changes in overall filament behavior. The most dramatic example of this conformational selectivity is seen for sAB-4. Evidently, sAB-4 competes successfully for native interactions in the F-actin structure, leading to a filament break. The bundling or crosslinking mechanism of sAB-27 is unclear, but may involve an extended binding-site that can bridge two closely-spaced actin filaments. Finally, sAB-19 interactions appear to be the most conformationally “neutral,” since it can bind to filaments and cap the pointed end, without otherwise affecting filament structure or organization. Thus, the three sABs described here have three structurally distinct modes of binding to actin filaments, even though they all target the same pointed end of actin near the DNase I binding loop.

### Use of artificial ABPs in live cells

The impact of artificial ABPs for dissecting and engineering actin biology is dramatically increased if they can be introduced into live cells. This is potentially a huge bottleneck since introducing large proteins (sABs are about 55 kDa) into cells with intact function can be problematic. However, in a recent breakthrough this barrier was overcome using a novel receptor-mediated delivery system [15]. We characterized the effects of the three pointed-end actin binding sABs (sAB-4, sAB-19 and sAB-27) when introduced into the cytosol of live cells. Each of the sABs was fluorescently labeled and conjugated to SP through an engineered cysteine mutation at position 121 of its heavy chain. The results confirmed that the properties observed in the *in vitro* characterizations are recapitulated in the cytosol of cells based on the cell imaging analyses [15]. Further, the extensive bundling produced by sAB-27 was shown to have a concentration dependent effect on cell viability leading to cell death after a 30 h at concentrations as low as 20 nM. Clearly, the disruption of the actin cytoskeleton at the level produced by sAB-27 is lethal to the cell making it a potential therapeutic agent in the context of its internalization via the SP/NK1 pathway, which demonstrates higher specificity for cancer versus normal tissue. Thus, we envision that sABs will prove to be useful reagents for remodeling cellular functions in live cells as desired.

## Materials and Methods

### Reagents

Actin was purified from chicken muscle acetone powder [19]. Actin was typically prepared as a 10 µM stock with 33% tetramethylrhodamine (TMR) G-actin [20]. In procedures where F-actin was immobilized on surfaces, filaments were polymerized with 10% biotin G-actin. For phage selection, actin biotinylation at cys374 was performed with the cleavable linker (N-(6-(Biotinamido)hexyl)-3′-(2′-pyridyldithio)-propionamide EZ-Link Biotin-HPDP, using previously described procedures [21]. Actin was prepared either in F-buffer (5 mM Tris•HCl pH 7.4, 50 mM KCl, 2 mM MgCl_2_, 1 mM ATP, 10 mM DTT) or in G-buffer (2 mM Tris•HCl pH 8.0, 0.2 mM CaCl_2_, 0.2 mM ATP). Full-length gelsolin was the generous gift of John Dawson (University of Guelph, Ontario, Canada).

### Selection of sABs against F-actin

We used established library construction and manipulation protocols as previously described [13], [14]. Briefly, we performed three rounds of selection at room temperature using the magnetic bead method with minor modifications [22]. In the first round, 100 µL of 1 µM biotinylated actin in G-buffer was immobilized on an excess of streptavidin magnetic beads and washed three times with nonreducing F-buffer. The beads were incubated with ∼10^13^ cfu phages for 15 min in 1 mL nonreducing F-buffer (5 mM Tris•HCl pH 7.4, 50 mM KCl, 2 mM MgCl_2_, 1 mM ATP) supplemented with 0.1 mg/mL BSA. The solution was discarded and the beads were washed three times with nonreducing F-buffer. Phages were eluted with F-buffer containing 100 mM DTT for 10 min to cleave the disulfide link to the resin. To decrease the background streptavidin binding for the subsequent rounds of selection, the recovered phages were amplified and incubated with streptavidin beads for 15 min, after which the beads were discarded. We used the same procedures for the second and third rounds, except we used 100 nM biotinylated G-actin in the second round and 10 nM biotinylated G-actin in the third round.

After selection, we isolated and grew individual clones for phage ELISA tests [23] to identify genuine pointed-end binding proteins. We prepared two parallel ELISA Maxisorp plates: 1) actin in F-buffer, and 2) actin blocked with DNase I in F-buffer. These plates were treated with individual actin-binding phages for 15 min and developed as described [23]. We identified seventeen phages with a greater than twofold reduction in ELISA signal from the DNase I plate relative to the actin-only plate, and sequenced each of these. Out of the seventeen clones selected, ten were unique. The sABs were expressed and purified as described [14], [24].

### Epifluorescence and TIRF microscopy

All epifluorescence images of actin filament bundles were acquired on a Zeiss Axiovert 200 using an Andor Luca EMCCD. TIRF images were collected on a custom-built objective-type TIRF microscope as previously described [25]. A 100×, 1.45 NA objective (Olympus) and an EMCCD camera (iXon, Andor Technology) were used for TIRF.

### Nucleation assay

We constructed flow cells from two pieces of double-sided tape adhered to a microscope slide with a coverslip placed on top, forming a chamber with a 10 µL volume. This flow cell was incubated with 1 mg/mL neutravidin in buffer AB (25 mM KCl, 25 mM imidazole•HCl pH 7.5, 1 mM EGTA, 4 mM MgCl_2_, 10 mM DTT), followed by blocking with 1 mg/mL BSA in AB buffer. Flow cells were then washed with 10% (w/v) Triton X-100, followed by an extensive TIRF buffer wash. G-actin (15 µM stock, 10% biotinylated, 33% TMR) was diluted to 1 µM in TIRF buffer (10 mM imidazole•HCl pH 7.0, 100 mM KCl, 3 mM MgCl_2_, 1 mM EGTA, 50 mM DTT, 50 µM CaCl_2_, 1% (w/v) methylcellulose-CP15, 0.5% (w/v) Triton X-100, 4.3 mg/mL glucose oxidase, 0.7 mg/mL catalase, 4.5 mg/mL glucose, 0.5% (v/v) BME, and 2 mM ATP). The actin solution was perfused into the chamber for 2 minutes, then washed with TIRF buffer. TIRF Images were acquired 5 min after initiation of polymerization, and filaments were counted.

### Direct visualization of end capping by sAB-19

We labeled sAB-19 at a single cysteine mutation (A121C) with a 5-fold molar excess of Cy5-maleimide (GE Healthcare) overnight in PBS. Excess dye was removed by overnight treatment of the sAB-19 solution with Bio-Beads SM-2 (Bio-Rad) to adsorb the hydrophobic dye. We immobilized actin filaments to a coverslip as follows. A microscope flow cell was incubated with 1 mg/mL neutravidin, followed by blocking with 1 mg/mL BSA. F-actin (200 nM; 33% TMR, 10% biotinylated) was prepared in AB (25 mM imidazole•HCl pH 7.4, 25 mM KCl, 4 mM MgCl_2_, 1 mM EGTA, 10 mM DTT). The actin solution was added to the flow cell for 2 min, followed by an AB wash. Cy5-labeled sAB-19 was diluted into AB with the oxygen scavenging system (glucose oxidase, catalase, glucose, and BME, as in the nucleation assay) and added to the chamber. We acquired an initial actin image in the TMR channel, followed by a movie of the sAB-19 binding events (0.2 s exposure for 200 frames). Images were merged in ImageJ.

### Seeded polymerization assay with capping protein for sAB-19

We produced seeds with a free pointed end by polymerizing 5 µM G-actin (33% TMR-labeled, 10% biotinylated) in the presence of 500 nM mouse capping protein (Cp) [26] by the addition of polymerization salts (2 mM final ATP, 1 mM final MgCl_2_ 50 final mM KCl). After ∼18 h of polymerization, sAB-19 was added (0–10 µM), followed by 2 µM G-actin (33% TMR-labeled) and polymerization salts. After an additional ∼18 hours to allow for complete polymerization off of the slow-growing pointed end, the filaments were diluted 100× into F-buffer (plus oxygen scavengers). Diluted filaments were attached to the surface of flow cells and visualized in TIRF as described above for the nucleation assay.

### Actin bundling by sAB-27

F-Actin (10 µM, 33% TMR-labeled) was diluted 100× into F-buffer with sAB-27 (10 µM to 10 nM) and oxygen scavengers, washed into a flow cell, and visualized in epifluorescence. For electron microscopy, we applied actin filaments alone (5 µM in F-buffer), or filaments incubated with sAB-27 (1 µM, for 5 s), to holey carbon film grids (Quantifoil, Cu 400 mesh). The grids were washed twice with 0.1 M NaCl, and then stained with 1% uranyl acetate. Electron Micrographs were obtained on a FEI Tecnai F30 transmission electron microscope at 75000X.

### Pyrene-actin depolymerization assay for sAB-4

Depolymerization was initiated by dilution of 10 µM F-actin (10% pyrene labeled on Cys374) to a final concentration of 190 nM in F-buffer with sAB-4 (1 µM to 10 nM). Pyrene-fluorescence was measured at 20°C in a FluoroMax 3 spectrofluorimeter after 8–10 s of mixing by inversion in a conventional 1.7 mL cuvette. The first measurement occurs 15 s after dilution. Excitation and emission wavelengths were 360 and 386 nm, respectively. This procedure was adapted from [27].

### Visualization of actin severing by sAB-4

Glass flow cells were treated with neutravidin and blocked as described in the nucleation assay. F-actin (200 nM, 2% biotinylated) in F-buffer was added to the flow cells for 2 minutes. Flow cells were washed with F-buffer plus oxygen scavengers and imaged in TIRF. We added sAB-4 (0.1 to 0.7 µM in F-buffer plus oxygen scavengers) to the chamber after the start of the movie to observe the entire timecourse. Movies were acquired with a 0.2 s exposure for 200 frames.

## Supporting Information

Supporting Information S1 Identification of a specific actin-binding motif.
(0.03 MB PDF)

Figure S1 Gel filtration of actin and actin:sAB-4 complex. (A) Gel Filtration of 95 μM G-actin was performed on an S300 (26/60; GE Healthcare) column, to distinguish the monomeric peak. Monomeric fractions were pulled and concentrated to 10 μM and re-run on the S300 column (red). A small non-monomeric peak is still detectable (∼4200mL). sAB-4 and G-actin were complexed 1:1 (molar ratio) for 5 minutes, spun at 13.2K Rpm for 10 minutes, then loaded on the column. A shift in the peak is detectable indicating that sAB-4 and actin are complexed. (B) 15% SDS-PAGE of the actin only fractions 54-62. (C) 15% SDS-PAGE of the sAB-4:actin complex fractions 54-62. An obvious shift to the left (earlier fractions) sAB-4:actin complex fractions is seen.
(4.93 MB EPS)

Figure S2 Maltose binding sAB in the presence of F- and G-actin. F-actin (10 μM) was polymerized for 90 minutes and overnight in the presence of varying concentrations of Maltose binding protein sAB. No effect was seen on polymerization. 10 μM G-actin was also put in the presence of Maltose binding protein sAB for 5 minutes, then polymerization was induced for 90 minutes and overnight. No effect on actin polymerization was seen. Scale bar  =  1 μm.
(0.87 MB EPS)
