# Supplementary material for: Characterization of Engineered Actin Binding Proteins That Control Filament Assembly and Structure
Source: PLoS One. 2010 Nov 12;5(11):e13960. doi: 10.1371/journal.pone.0013960 (PMC2980482; doi:10.1371/journal.pone.0013960)
Supplement: Supporting Information S1 — Identification of a specific actin-binding motif. (0.03 MB PDF) [file pone.0013960.s001.pdf]

Supplementary Information 1: Identification of a specific actin-binding motif

Our goal is to estimate the probability of finding the sequence motif [RK][FY][RK][FY] in the CDR-H3 loops 4 times out of 10 (see Fig. 1c) by chance alone. To begin, we require an estimate of the fraction of our library that contains the motif.

In our phage display library, CDR-H3 was designed with a mixture of 20% Y, 15% S, 15% G, and 3% all other amino acids, excluding C. The loop length is allowed to vary between 10 - 22 residues (TONY: do we know the proportions of each loop length?). Note that the library size, 10^{13} phages, is much smaller than the total sequence space. The probability of finding the sequence [RK][FY][RK][FY] in a contiguous stretch of four residues is (0.06)(0.23)(0.06)(0.23) = 1.9×10^{-4}. For loop lengths of 10 residues, there are 7 independent ways of placing a stretch of four residues; for loop lengths of 22 residues, there are 19 placements of four residues. Thus, the probability of drawing our sequence from the library ranges from:

$P_s = 1 - (1 - 1.9 \times 10^{-4})^7 = 1.3 \times 10^{-3}$ (n = 10)

to:

$P_s = 1 - (1 - 1.9 \times 10^{-4})^{19} = 3.6 \times 10^{-3}$ (n = 22)

depending on the loop length, n. These probabilities assume the libraries contain exclusively loops of the given length.

Next, given these odds, we estimate the binomial probability of drawing these sequences 4 times out of 10 trials by chance. These probabilities range from:

$P(x >= 4 \mid n = 10) = 5.9 \times 10^{-10}$

to:

$P (x >= 4 \mid n = 22) = 3.4 \times 10^{-8}$

We conclude that the sequence motif presented here is a particularly favorable actin binding motif, unlikely to appear by chance alone.
